# A Dose Escalation Study of Trientine Plus Carboplatin and Pegylated Liposomal Doxorubicin in Women With a First Relapse of Epithelial Ovarian, Tubal, and Peritoneal Cancer Within 12 Months After Platinum-Based Chemotherapy

**DOI:** 10.3389/fonc.2019.00437

**Published:** 2019-05-24

**Authors:** Yu-Fang Huang, Macus Tien Kuo, Yi-Sheng Liu, Ya-Min Cheng, Pei-Ying Wu, Cheng-Yang Chou

**Affiliations:** ^1^Department of Obstetrics and Gynecology, National Cheng Kung University Hospital, College of Medicine, National Cheng Kung University, Tainan, Taiwan; ^2^Department of Molecular Pathology, The University of Texas MD Anderson Cancer Center, Houston, TX, United States; ^3^Department of Medical Imaging, College of Medicine, National Cheng Kung University Hospital, National Cheng Kung University, Tainan, Taiwan

**Keywords:** ovarian cancer, trientine, carboplatin, pegylated liposomal doxorubicin, adverse effect, ceruloplasmin, copper, human copper transporter 1

## Abstract

**Background:** Epithelial ovarian cancer (EOC) is the leading cause of gynecological cancer-related deaths worldwide. Preclinical studies found that copper-lowering agents could re-sensitize platinum-resistant cancer cells by enhancing the human copper transporter 1 (hCtr1)-mediated uptake of platinum. In the clinic, re-sensitization of platinum-resistance in relapsed EOC has been discovered by the application of trientine plus platinum (NCT01178112). However, no pharmacokinetic data of trientine has been reported in cancer patients.

**Purpose:** Our study aimed to explore the safety and activity of trientine combined with carboplatin and pegylated liposomal doxorubicin (PLD) in patients with EOC, tubal, and peritoneal cancer who experienced disease progression during platinum-based chemotherapy or showed relapse <12 months after completing first-line chemotherapy. Also, we aimed to demonstrate pharmacokinetic parameters and to discover potential biomarkers in our EOC patients.

**Methods:** In this dose escalation study, 18 Asian patients in six dosing cohorts received fixed doses of carboplatin (AUC 4) and PLD (LipoDox®, TTY Biopharm Co. Ltd., Taipei, Taiwan) (40 mg/m^2^, day 1 per 4-week cycle), and escalated daily trientine doses (range: 300–1800 mg; initiated 7 days before the 1st combination cycle) according to a 3 + 3 design.

**Results:** No dose-limiting toxicity or treatment-related death was observed. Four patients (22.2%) developed grade 3 drug-related adverse events (AEs), whereas no grade 4 AEs were encountered. Anemia and grade 2 dizziness were the most common hematological toxicity and neurotoxicity, respectively. In a pharmacokinetics comparison with healthy volunteers in the literature, our patients achieved greater absorption after oral trientinem, and more rapid elimination of triethylenetetramine dihydrochloride at high doses. The clinical benefit rate was 33.3 and 50.0% in the platinum-resistant and the partially platinum-sensitive group, respectively. A high baseline serum iron level and low serum copper level might help differentiate subgroups of patients with different clinical responses. Nevertheless, no associations of the clinical response with the levels of serum hCtr1, ceruloplasmin, or copper were observed.

**Conclusion:** Combination therapy with carboplatin, trientine, and PLD was well-tolerated and safe. Our results encourage the development of a future phase II trial.

**Clinical trial registration:** ClinicalTrials.gov # NCT03480750.

## Introduction

Epithelial ovarian cancer (EOC) is the leading cause of gynecological cancer-related deaths in the United States (US) and the second leading cause in other countries (1–3). Most EOC patients will eventually progress through several lines of chemotherapy and/or investigational treatment options. A reduced drug transport capacity is a complex and substantial mechanism of platinum-resistance in a tumor (4, 5), which presents a significant challenge to EOC treatment. The human copper transporter 1 (hCtr1) regulates platinum uptake by tumor cells (6–9). Increased hCtr1 expression in EOC was associated with a favorable response rate and better progression-free survival (PFS) and overall survival (OS) (10).

Copper-lowering agents such as triethylenetetramine dihydrochloride (TETA, trientine) and D-penicillamine have long been approved to treat copper metabolism disorders such as Wilson's disease (WD). The adverse effects (AEs) associated with these drugs are well-known. Copper-lowering agents were shown to re-sensitize platinum-resistant cancer cells by enhancing the hCtr1-mediated uptake of platinum (11–14). Subsequently, a dose-escalation study of carboplatin at a dosage of area under the curve (AUC) 4-6, together with daily trientine at 2,000–3,000 mg beginning 7 days before carboplatin administration, reported that no dose-limiting toxicity (DLT) or treatment-related deaths in patients with advanced solid malignancies (NCT01178112) (15, 16). Notably, one of eight patients with platinum-resistant EOC after ≥3 lines of chemotherapy achieved a partial response (PR), while two others achieved stable disease (SD) for ≥6 months. This suggests that this treatment strategy may achieve partial re-sensitization of platinum resistance in EOC. Moreover, those investigators observed a statistical significance between reduced serum ceruloplasmin levels (5–15 mg/dL) and clinical benefit in patients (15, 16). However, the small patient population in that study precludes definitive conclusions.

Some studies have investigated the use of pegylated liposomal doxorubicin (PLD) in recurrent EOC patients (17–19). A randomized trial of PLD for recurrent platinum-sensitive or -resistant EOC reported a tumor response rate of 19.7% (17). Taiwanese Gynecologic Oncology Group et al. (19) reported an overall response rate of 23.1% among 29 patients with platinum-resistant/-refractory disease in a multicenter phase II trial of PLD (LipoDox®, TTY Biopharm Co. Ltd., Taipei, Taiwan). Thus, our study included LipoDox® in conjunction with carboplatin and trientine. However, the optimal dose of trientine in such combination in EOC patients has never been reported. Therefore, we performed an EOC mouse xenograft model, which showed that tumor-inhibiting effects of carboplatin plus PLD could be reinforced by advancing the timing of oral trientine (Supplementary Table 1 and Supplementary Figure 1). Based on these encouraging results, we conducted the present study to include LipoDox® in conjunction with carboplatin and trientine in patients with platinum resistance. Additionally, we enrolled patients with early relapsed EOC, which occurs 6–12 months after the completion of primary chemotherapy (i.e., partially platinum-sensitive) and exhibits potential platinum-resistance.

In this context, our study focused on EOC patients, including patients with tubal cancer (TC), or primary peritoneal serous carcinoma (PPSC) who exhibited cancer progression during primary chemotherapy or soon after completing first-line chemotherapy. Our primary aim was to describe the safety of a combination regimen of trientine with carboplatin and PLD, as well as the optimal biologic dosage of trientine in these patients. Secondarily, the study aimed to describe pharmacokinetics (PK), and to correlate clinical responses or outcomes with the expressions of ceruloplasmin, copper, or other possible biomarkers, such as hCtr1.

## Patients and Methods

### Participants

The clinical research protocol was approved by the National Cheng Kung University Hospital (NCKUH) Institutional Review Board. Each patient provided written consent in accordance with the Declaration of Helsinki. Female patients aged 20–75 years with histologically proven EOC/TC/PPSC were considered eligible for the trial if their malignancies had relapsed within 12 months after primary platinum-based chemotherapy or no available therapy had prolonged survival by at least 3 months.

#### Patient Inclusion Criteria

All patients had undergone comprehensive staging or cytoreductive surgery followed by postoperative platinum-based chemotherapy, with or without bevacizumab. Tumors were staged according to the criteria of the International Federation of Gynecology and Obstetrics (FIGO). Other patient eligibility criteria included the presence of measurable or evaluable disease, an Eastern Cooperative Oncology Group (ECOG) performance status of ≤2, adequate bone marrow function (absolute neutrophil count ≥1,500/μL, hemoglobin level ≥9.0 g/dL, and platelet count ≥100,000/μL), a serum creatinine level ≤1.5 mg/dL, or calculated creatinine clearance of ≥50 mL/min, a total serum bilirubin level ≤2.0 mg/dL, and an aspartate transaminase or alanine transaminase level ≤5× the upper normal limit. Patients who could potentially become pregnant were required to use an effective method of birth control prior to study entry and for the duration of their participation in the study.

#### Patient Exclusion Criteria

The following patients were excluded from the study: those receiving concurrent chemotherapy, those who had not recovered from surgery within 4 weeks of the study, those with a clinically significant medical condition that could be aggravated by treatment or could not be controlled, those with medical and/or psychiatric problems of sufficient severity to limit full compliance with the study or expose patients to undue risk, those with known anaphylactic responses or severe hypersensitivity to study drugs or their analogs, pregnant, or lactating women, patients with any evidence of dysphagia or intestinal obstruction and patients who were unwilling or unable to provide informed consent.

### Study Design

This phase I trial was performed using a 3 + 3 design. An additional three patients were allowed for the safety assessments as required. This phase aimed to expand the maximum tolerated dose (MTD). Recruitment of eligible patients was limited to within 3 years due to the sponsorship period, and the participants would be individually followed for at least 18 months after completion of the combination therapy.

We escalated the fasted oral daily dose of trientine from 300 mg to a maximum of 1,800 mg in six dosing cohorts. Trientine therapy was initiated 7 days prior to the 1st combination therapy cycle. Each patient started receiving an intravenous infusion of carboplatin at AUC 4 over a 2-h period and PLD (Lipo-Dox®) at 40 mg/m^2^ over a 1.5 h period on day 1 of each 28 days cycle for a maximum of six cycles as long as the patient did not exhibit evidence of tumor progression or prohibitive toxicity. The daily dosage of trientine was adjusted based on uncorrectable drug-related AEs at the treating physician's discretion, according to the study protocol. This involved a decrease to 900 mg if the treatment dose was between 1,200 and 2,400 mg or to 1,200 mg if the treatment dose was >2,400 mg. Chemotherapeutic doses were reduced by a maximum of 25% in response to ≥grade 3 hematological or non-hematological toxicities according to the physician's discretion. There were restrictions on the use of calcium or magnesium antacids or iron to prevent interference with the absorption of trientine.

### Safety

AE severity was graded according to the Common Terminology Criteria for Adverse Events, version 4.03 (20). All patients who received at least one dose of any of the study agents were considered evaluable for safety. One patient declined all of the study drugs after enrollment and was deemed ineligible for the analysis. Hence, one additional patient was included at the same trientine dosage level (600 mg daily). Patients receiving trientine treatment were monitored to evaluate the serum levels of ceruloplasmin, copper, and iron.

### DLTs and MTD

DLTs were defined as drug-related grade 3 non-hematological adverse events (except alopecia or non-drug-related nausea/vomiting) lasting for >7 days, grade 4 hematological events >7 days, febrile neutropenia, or thrombocytopenia (platelet count <25,000/μL) during the first treatment cycle or a need to delay the start of the second treatment cycle by >2 weeks. The MTD was defined as the level below the dose at which ≥33% patients experienced a drug-related DLT during the first treatment cycle.

### Pharmacokinetics Study

Blood samples were collected prior to the start of the first trientine dose in cycle 1 (i.e., baseline), and at 10, 30 min, and 1, 1.5, 2, 4, 6, and 24 h after the first oral trientine dose. Twenty-four-hour urine samples were collected on days 1, 7, 14, and 64 after start of the first oral trientine. The plasma levels of TETA and its two major metabolites, N1-acetyltriethylenetetramine (MAT), and N1,N10-diacetyltriethylenetetramine (DAT), were quantified by liquid chromatography/tandem mass spectrometry and analyzed in accordance with the principles of Good Laboratory Practice.

### Biomarker Examination

Blood samples were collected prior to the start of the first trientine dose in cycle 1 (i.e., baseline), on the day prior to each cycle of the combination therapy, and 1 month after the end of the combination therapy. Urine samples were collected prior to the start of the first trientine dose in cycle 1 (i.e., baseline), and on days 7, 14, and 64 after start of the first oral trientine.

Serum ceruloplasmin, the major copper-carrying protein in the blood, was measured using an immunonephelometry assay (normal range: 22–55 mg/dL). Serum and urine copper levels were measured by flam atomic absorption spectrometry (normal range: 80–155 μg/dL and 15–50 μg/24 h, respectively). Serum iron levels were measured using a colorimetric assay (normal range: 37–145 μg/dL).

An enzyme-linked immunosorbent assay kit (MyBioSource, San Diego, CA, USA; Catalog No MBS885339; sensitivity: 0.225 ng/mL) was used in duplicate to measure hCtr1 levels. Any test with an intra-assay coefficient of variation (CV) >10% was repeated. The average intra- and inter-assay CVs were <10 and 12%, respectively.

### Efficacy

Tumor responses were evaluated using the Response Evaluation Criteria in Solid Tumors, version 1.1 (21) or the Gynecologic Cancer Intergroup definition for carbohydrate antigen (CA) 125 progression (22). All patients who received one cycle of the three study agents were considered evaluable for efficacy. All pathological diagnoses were centrally reviewed at NCKUH. A follow-up of at least 18 months was planned for all patients.

A clinical benefit was defined as a complete response (CR), PR, or SD for a duration of ≥4 months. Patients who were removed from the study before the first scheduled restaging workup due to progression, serious drug-related adverse events, or any other reasons were considered treatment failures and arbitrarily designated as having a disease progression of 21% in a waterfall plot to determine the best tumor responses. PFS was defined as the time interval from the date of initial treatment to the first objective documentation of disease progression or the time of death if applicable. Patients who remained alive without disease progression at the last follow-up on May 31, 2018 were censored as of that date. OS was estimated from the date of initial treatment until the date of death or censoring on May 31, 2018.

### Statistical Analysis

Data were analyzed using SPSS (version 17.0; SPSS Inc., Chicago, IL, USA). Interval variables are presented as the means ± standard errors of the means or as medians ± interquartile ranges. Differences between groups were evaluated using the Mann–Whitney *U*-test. Frequency distributions between categorical variables were compared using the Pearson chi-square and Fisher's exact tests. The urine concentrations of TETA, MAT, and DAT at six dose levels were compared using the Kruskal–Wallis test. Survival was estimated using the Kaplan–Meier method and compared using log-rank tests. A *P* < 0.05 (two-sided) was considered significant.

## Results

### Patient Demographics

**Figure 1:**
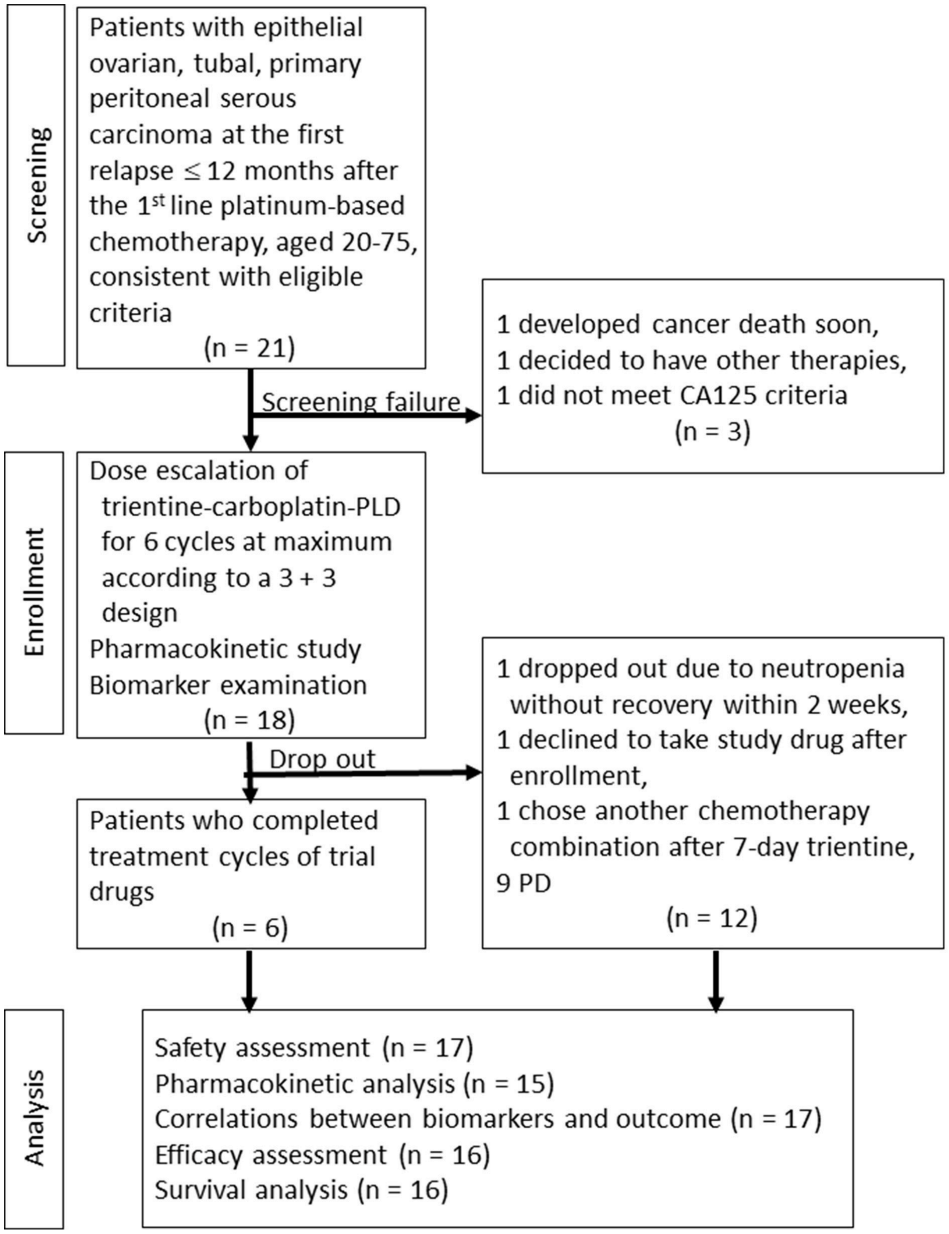
Study flow chart.

Between September 1, 2012 and October 30, 2015, 18 patients (median age, 53.5 years; range, 44–74 years) were enrolled. The study flow chart is shown in Figure 1. One patient receiving 600 mg/day declined the trial drugs and did not proceed with treatment. Therefore, an additional participant was included at the same dose level. The patients' baseline characteristics are shown in Table 1. The median follow-up period was 17.4 months (range 2.7–58.8 months).

**Table 1:** Baseline characteristics and treatment outcome of the 18 study participants.

**No**	**Dose level of trientine**	**Cancer type**	**Histology**	**First-line chemotherapy**	**PFI (months) after first-line chemotherapy**	**Baseline serum copper level (μg/dL)**	**Baseline ceruloplasmin level (mg/dL)**	**Trial cycles**	**RECIST 1.1 Best Response**	**PFS (months)**	**OS (months)**
1	300	Ovarian	Mucinous	Paclitaxel/carboplatin	2.6	118.0	42.0	3	PD	2.3	3.2
2	300	Ovarian	Serous	Paclitaxel/carboplatin	0.4	92.0	30.3	1	PD	1.0	2.7
3	300	Ovarian	Mixed serous and clear cell	Paclitaxel/carboplatin	11.5	188.0	65.5	6	CR	46.3	58.8
4	600	Ovarian	Serous	Paclitaxel/carboplatin	9.3	88.0	30.4	5	PD	4.3	10.2
5	600	Ovarian	Serous	Paclitaxel/carboplatin	6.0	110.0	45.3	1	PD	1.1	22.6
6*	600	Ovarian	Serous	Paclitaxel/carboplatin	7.0	102.0	47.5	0	-	−	−
7	600	Ovarian	Mixed endometrioid and clear cell	Paclitaxel/carboplatin/bevacizumab	7.1	176.0	53.9	3	PD	7.9	9.8
8	900	Ovarian	Clear cell	Paclitaxel/carboplatin	10.8	289.0	76.7	2	PD	1.8	5.8
9	900	Ovarian	Serous	Paclitaxel/carboplatin	10.7	103.0	36.3	6	CR	9.5	20.7
10	900	Ovarian	Mucinous	Paclitaxel/carboplatin	1.1	176.0	64.8	4	PD	4.6	5.4
11	1,200	Ovarian	Serous	Paclitaxel/carboplatin	4.2	74.0	24.3	6	SD	26.6	34.5
12	1,200	Ovarian	Clear cell	Paclitaxel/carboplatin/bevacizumab	11.9	122.0	33.2	5	PD	4.4	7.2
13	1,200	Ovarian	Clear cell	Paclitaxel/carboplatin/bevacizumab	0.7	184.0	43.3	6	SD	9.2	21.2
14	1,500	Ovarian	Serous	Paclitaxel/carboplatin	10.0	113.0	38.0	4	PR	12.7	32.8
15	1,500	Peritoneal	Serous	Paclitaxel/carboplatin/bevacizumab	3.3	87.0	30.7	3	PD	2.1	20.3
16	1,500	Ovarian	Clear cell	Paclitaxel/carboplatin/bevacizumab	10.7	136.0	41.0	6	SD	8.9	14.4
17	1,800	Ovarian	Serous	Paclitaxel/carboplatin	6.1	123.0	40.5	6	PR	12.5	32.9
18**	1,800	Ovarian	Serous	Paclitaxel/carboplatin	5.9	76.0	29.5	0	-	−	−

FIGO, the International Federation of Gynecology and Obstetrics; OS, overall survival; PFS, progression free survival; PFI, progression free interval; RECIST, Response Evaluation Criteria In Solid Tumors.

* *No. 6 participant declined the trial drugs, did not proceed with treatment and died of disease progression 1 year later*.

** *No. 18 participant chose another chemotherapy combination after 7-day trientine, not due to treatment failure and died of disease progression 2 years later*.

Of these patients, one each (5.6%) was classified as having FIGO stages IA, IC, IIC, and IIIB; 10 (55.6%) and four (22.2%) were in stages IIIC and IV, respectively. Seventeen patients (94.4%) were diagnosed with EOC; one (5.6%) was diagnosed with PPSC. A majority of patients (*n* = 10, 58.8%) had serous histology. Four (22.2%) had clear cell carcinoma (CCC), two (11.1%) had mucinous histology, one (5.6%) had mixed serous histology and CCC, and one (5.6%) had mixed endometrioid histology and CCC.

### Safety Assessment

Seventeen patients were evaluable for toxicity (Table 2). Sixty-eight cycles of therapy were administered during this clinical trial. No DLTs or treatment-related deaths were reported. Commonly observed grade 2 AEs included anemia (*n* = 11, 64.7%), leukopenia (*n* = 7, 41.2%), anorexia (*n* = 7, 41.2%), vomiting (*n* = 4, 23.5%), nausea (*n* = 3, 17.6%), alopecia (*n* = 3, 17.6%), neutropenia (*n* = 2, 11.8%), and paresthesia (*n* = 1, 5.9%). One patient (5.9%) experienced grade 2 dizziness, a trientine-associated AE, which caused significant discomfort and prompted a dose reduction from 1,200 to 900 mg in the following cycles, according to the study protocol. grade 3 adverse events reported in this trial included anemia (*n* = 2, 11.8%), diarrhea (*n* = 2, 11.8%) and neutropenia, thrombocytopenia, vomiting, hyponatremia, and hyperkalemia (*n* = 1 each, 5.9%). The only grade 4 treatment-related adverse event was thrombocytopenia, which recovered within 7 days (*n* = 1, 5.9%). Both patients who received trientine at a divided daily dose of 1,800 mg tolerated the treatment well.

**Table 2:** Frequency of adverse effects by 6 dose levels of trientine (*n* = 17).

**Level**	**I (*****n*** **= 3)**			**II (*****n*** **= 3)**			**III (*****n*** **= 3)**				**IV (*****n*** **= 3)**			**V (*****n*** **= 3)**			**VI (*****n*** **= 2)**		
Trientine (mg/day)	300			600			900				1200			1500			1800		
LipoDox 40 mg/m^2^ and carboplatin AUC 4 q4w																			
**CTCAE v4.0 grade**	**1**	**2**	**3**	**1**	**2**	**3**	**1**	**2**	**3**	**4**	**1**	**2**	**3**	**1**	**2**	**3**	**1**	**2**	**3**
Leukopenia	0	1	0	0	1	0	0	2	0	0	1	1	0	2	2	0	0	0	0
Neutropenia	0	1	0	0	0	0	0	0	0	0	0	1	0	0	0	1	0	0	0
Anemia	3	2	0	1	1	0	2	3	1	0	2	2	1	3	2	0	1	1	0
Thrombocytopenia	1	0	0	1	0	0	1	1	1	1	0	0	0	0	0	0	0	0	0
Nausea	2	2	0	2	0	0	2	1	0	0	3	1	0	3	0	0	1	0	0
Vomiting	1	2	0	2	0	0	2	0	1	0	3	1	0	3	0	0	1	0	0
Constipation	1	0	0	2	0	0	2	0	0	0	1	1	0	1	0	0	0	0	0
Diarrhea	1	0	0	1	0	1	1	1	1	0	0	0	0	0	0	0	0	0	0
Mucositis	0	0	0	1	0	0	1	0	0	0	2	0	0	3	1	0	1	0	0
Numbness (Paresthesia)	1	0	0	2	1	0	1	0	0	0	1	0	0	2	0	0	1	0	0
Dizziness	0	0	0	1	0	0	1	0	0	0	1	0	0	1	1	0	1	0	0
Fatigue	1	1	0	0	0	0	0	1	0	0	3	0	0	2	0	0	0	0	0
Anorexia	2	0	0	0	0	0	2	3	0	0	3	2	0	3	1	0	1	1	0
Hand-foot syndrome	0	0	0	0	0	0	1	0	0	0	0	0	0	1	1	0	1	0	0
Skin rash	0	0	0	0	0	0	0	0	0	0	1	0	0	3	1	0	0	0	0
Alopecia	0	1	0	0	0	0	0	1	0	0	2	0	0	1	1	0	2	0	0
Fever	0	0	0	0	0	0	1	1	0	0	0	0	0	0	0	0	0	0	0
Hyponatremia	1	0	0	0	0	0	1	0	1	1	1	0	0	0	0	0	0	0	0
Hyperkalemia	0	0	0	0	0	0	0	1	1	0	0	0	0	0	0	0	0	0	0
AST increased	3	0	0	2	0	0	2	0	0	0	1	0	0	1	0	0	1	0	0
ALT increased	3	0	0	1	0	0	1	0	0	0	1	0	0	1	0	0	0	0	0
ALK-P increased	2	1	0	1	0	0	1	0	0	0	1	0	0	1	0	0	0	0	0

*AST/GOT, Aspartate aminotransferase; ALT/GPT, Alanine aminotransferase; ALK-P, Alkaline phosphatase*.

### Pharmacokinetics

Fifteen patients (83.3%) were included in the pharmacokinetic study. The plasma elimination half-lives (T_1/2_), the time to reach the maximum concentration (T_max_), the maximum concentration (C_max_), and the area under the curve during 24 h (AUC_0−24_) values of TETA, MAT, and DAT at different dose levels are listed in Table 3. For TETA, MAT, and DAT, the T_max_ occurred 0.5–6.0, 4.0–6.0, and 4.0–6.0 h, respectively, in patients with ovarian cancer, indicating relatively slow absorption rates in the gut. The C_max_ of TETA ranged from 1.9 to 29.4 mg/L, and we observed an increasing trend among patients with increasing doses of trientine. The C_max_ values of MAT and DAT ranged from 2.4 to 7.0 and 0.4 to 1.1 mg/L, respectively. The AUC_0−24_ values of TETA, MAT, and DAT ranged from 8.2 to 388.1, 29.6 to 87.8, and 5.3 to 16.3 mg × h/L, respectively.

**Table 3:** Pharmacokinetic parameters of TETA, MAT, and DAT at 6 dose levels in EOC/PPSC patients.

**Dose level**	**Dose of trientine (mg/d)**	**TETA**				**MAT**				**DAT**			
		**AUC_**0−24**_ (mg × h/L)**	**T_**1/2**_ (hours)**	**T_**max**_ (hours)**	**C_**max**_ (mg/L)**	**AUC_**0−24**_ (mg × h/L)**	**T_**1/2**_ (hours)**	**T_**max**_ (h)**	**C_**max**_ (mg/L)**	**AUC_**0−24**_ (mg × h/L)**	**T_**1/2**_ (hours)**	**T_**max**_ (hours)**	**C_**max**_ (mg/L)**
1	300	8.16 (8.12)	3.51 (2.53)	1.50 (1.00)	1.87 (0.86)	29.59 (32.89)	4.87 (0.00)	4.00 (4.50)	2.42 (2.96)	7.75 (4.75)	Undetectable	6.00 (2.00)	0.56 (0.54)
2	600	15.12 (4.12)	2.64 (0.00)	1.50 (1.00)	5.07 (4.24)	31.70 (0.98)	Undetectable	5.00 (2.00)	2.38 (0.04)	5.34 (0.81)	Undetectable	6.00 (0.00)	0.36 (0.06)
3	900	30.28 (45.49)	1.85 (2.86)	0.50 (0.50)	11.54 (8.22)	40.96 (71.21)	Undetectable	4.00 (2.00)	6.95 (3.81)	4.58 (12.52)	Undetectable	6.00 (0.00)	0.83 (0.73)
4	1,200	90.80 (56.45)	3.93 (2.16)	2.00 (0.50)	14.98 (6.03)	87.83 (99.23)	Undetectable	4.00 (2.00)	6.92 (7.96)	13.43 (15.63)	Undetectable	6.00 (0.00)	0.94 (1.22)
5	1,500	127.66 (31.35)	3.48 (0.00)	4.00 (3.00)	13.08 (14.02)	82.26 (58.94)	Undetectable	6.00 (0.00)	6.62 (4.71)	16.32 (11.99)	Undetectable	6.00 (0.00)	1.09 (1.06)
6	1,800	388.10 (0.00)	Undetectable	6.00 (0.00)	29.35 (0.00)	64.47 (0.00)	Undetectable	4.00 (0.00)	5.53 (0.00)	9.77 (0.00)	Undetectable	4.00 (0.00)	0.82 (0.00)

Data was presented as median (interquartile range).

*TETA, triethylenetetramine dihydrochloride; MAT, N1-acetyltriethylenetetramine; DAT, N1,N10-diacetyltriethylenetetramine; EOC, epithelial ovarian carcinoma; PPSC, primary peritoneal serous carcinoma*.

In all patients, the T_1/2_ ranged from 2.5 to 3.9 h, indicating rapid elimination of the parent compound. The T_1/2_ of MAT was longer than that of TETA at a trientine dose level of 300 mg/day. However, we could not obtain sufficient information regarding the T_1/2_ values of MAT and DAT at other dose levels. These values may have been beyond the detectable limits. In the urine, TETA is largely excreted in the form of the parental compound and MAT and DAT (13, 23–25). Notably, the urine concentrations of TETA, MAT, and DAT on days 7, 14, and 64 did not differ among the six trientine dosage levels (Table 4). Accordingly, we did not observe a dose-dependent relationship. No bioavailability or tissue distribution data were available.

**Table 4:** The concentration of 24-h urine TETA, MAT, and DAT on day 7, 14, and 64 at 6 dose levels in EOC/PPSC patients.

**24 h concentration**		**Dose level 1 (300 mg/day)**	**Dose level 2 (600 mg/day)**	**Dose level 3 (900 mg/day)**	**Dose level 4 (1,200 mg/day)**	**Dose level 5 (1,500 mg/day)**	**Dose level 6 (1,800 mg/day)**	***p*-value**
TETA (μM)	Day 1	2.47 (1.46)	11.06 (0)	14.90 (0.86)	79.87 (109.80)	38.75 (14.45)	118.13 (0)	0.1902
	Day 7	NA	NA	26.03 (25.41)	45.46 (48.52)	58.52 (7.47)	54.17 (0)	0.5724
	Day 14	NA	NA	20.65 (21.45)	40.06 (36.46)	17.25 (11.44)	22.68 (0)	0.9002
	Day 64	NA	NA	2.74 (0.68)	46.69 (9.60)	13.04 (8.93)	NA	0.1335
MAT(μM)	Day 1	57.55 (26.97)	67.83 (0)	179.25 (65.18)	316.33 (358.63)	149.2 (60.91)	225.65 (0)	0.1158
	Day 7	NA	NA	476.74 (340.31)	328.3 (292.98)	358.45 (288.52)	269.35 (0)	0.9250
	Day 14	NA	NA	577.57 (399.98)	249.64 (172.13)	183.34 (75.18)	283.96 (0)	0.2832
	Day 64	NA	NA	166.17 (5.71)	203.16 (79.69)	150.21 (13.69)	NA	0.5738
DAT(μM)	Day 1	22.14 (12.61)	9.42 (0)	31.14 (15.06)	58.36 (65.31)	25.72 (9.82)	29.22 (0)	0.7540
	Day 7	NA	NA	96.89 (62.92)	62.78 (51.83)	53.92 (34.51)	38.94 (0)	0.5899
	Day 14	NA	NA	110.86 (75.45)	50.28 (36.99)	46.12 (26.70)	51.05 (0)	0.5610
	Day 64	NA	NA	58.11 (14.18)	43.67 (8.33)	33.88 (15.30)	NA	0.3628

Data was shown as mean (SD) and data was analyzed by Kruskal-Wallis test.

*NA, not available; TETA, triethylenetetramine dihydrochloride; MAT, N1-acetyltriethylenetetramine; DAT, N1,N10-diacetyltriethylenetetramine; EOC, epithelial ovarian carcinoma; PPSC, primary peritoneal serous carcinoma*.

### Biomarker Examination

Patients' baseline serum ceruloplasmin levels are listed in Table 1. Treatment response-related changes in the serum levels of ceruloplasmin, copper, and iron, as well as the urine levels of copper and zinc are shown in Table 5. The mean baseline serum copper levels at trientine doses of 300, 600, 900, 1,200, 1,500, and 1,800 mg/day were 132.7 ± 49.7, 119.0 ± 39.1, 189.3 ± 93.7, 126.7 ± 55.1, 112.0 ± 24.5, and 99.5 ± 7.8 μg/dL, respectively, whereas the corresponding baseline ceruloplasmin levels were 45.9 ± 17.9, 44.3 ± 9.9, 59.3 ± 20.8, 33.6 ± 9.5, 36.6 ± 5.3, and 35.0 ± 7.8 mg/dL, respectively. The ceruloplasmin level nadir of each patient at each dose level did not reach 5–15 mg/dL. Compared to patients with progressive disease (PD), those who experienced clinical benefits from therapy had significantly higher baseline serum iron and lower serum copper levels after three cycles of treatment (*P* = 0.030 and *P* = 0.022, respectively). However, we did not discover significant correlations between the tumor response and the serum ceruloplasmin or hCtr1 levels or urine copper or zinc levels at any time point (data not shown). Furthermore, we did not observe correlations of the serum baseline levels of ceruloplasmin and copper with survival.

**Table 5:** Changes in serum levels of ceruloplasmin, copper, and iron, urine levels of copper and zinc related to treatment response.

	**PD (*n* = 9)**	**SD ≥ 4 M/PR/CR (*n* = 7)**	***p*-value**
**Serum Ceruloplasmin (mg/dL)**			
Baseline	45.25 (17.96)	42.44 (15.11)	1.000
After one cycle	46.76 (17.97)	36.25 (6.72)	0.315
After three cycle	49.10 (12.31)	33.47 (5.90)	0.100
Nadir	41.26 (15.78)	38.23 (16.90)	0.385
**Serum Copper (μg/dL)**			
Baseline	143.50 (68.99)	131.57 (41.84)	0.862
After one cycle	152.60 (41.74)	109.33 (28.25)	0.120
After three cycle	157.20 (40.07)	93.50 (26.04)	0.022
Nadir	128.50 (40.25)	106.43 (42.63)	0.247
**Serum Iron (μg/dL)**			
Baseline	35.50 (16.69)	62.83 (22.09)	0.030
After one cycle	49.80 (18.13)	64.00 (16.16)	0.234
After three cycle	55.75 (12.74)	60.00 (16.54)	1.000
Nadir	35.50 (16.69)	52.50 (15.54)	0.090
**Urine Copper**			
Baseline (μg/dL)	2.55 (2.74)	2.94 (2.82)	0.561
Day 14 (24 h) (μg)	83.74 (12.44)	94.22 (59.78)	1.000
Day 64 (24 h) (μg)	128.80 (–)	158.53 (129.56)	1.000
**Urine Zinc**			
Baseline (μg/dL)	49.70 (41.74)	55.07 (76.73)	0.654
Day 14 (24 h) (μg)	949.32 (896.66)	993.45 (379.33)	0.518
Day 64 (24 h) (μg)	910.00 (–)	1163.79 (929.82)	1.000

*Data was presented as mean (SD) and data was analyzed by Mann-Whitney U-test. CR, complete response; PR, partial response; PD, progressive disease; SD, stable disease*.

### Efficacy Assessment

**Figure 2:**
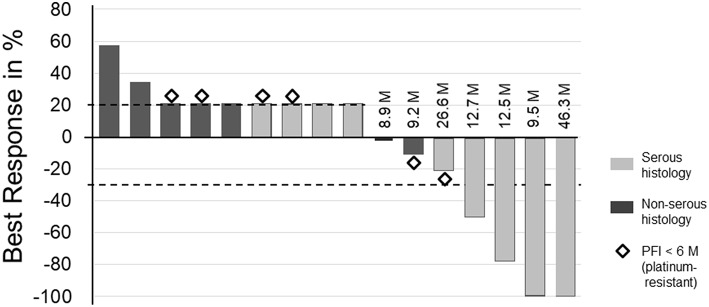
Best overall response from baseline per patient. The waterfall plot displays the best responses of 16 patients according to the RECIST version 1.1. The progression-free survival (PFS) duration (M, months) after trial agent administration are presented for each patient who achieved a complete response, partial response, or stable disease for ≥4 M. Diamonds indicate tumor responses in patients with a progression-free interval (PFI) <6 M prior to enrollment. Gray bars indicate patients with serous histology, and black bars indicate patients with non-serous histology. Patients with indicated 21% tumor increases either developed new tumor lesions, exhibited early tumor progression or withdrew early for other reasons. These patients were arbitrarily designated as having a disease progression of 21% or an actual tumor progression of 21%.

Sixteen patients were evaluable for efficacy (Figure 2). The clinical benefit rate was 43.8%. Two patients who declined any of the study agents after enrollment or declined the initial chemotherapy drug after a 7-day course of trientine were deemed ineligible for this analysis. Of the seven patients who were arbitrarily denoted as having a disease progression of 21%, two (28.6%) showed cancer progression before the first scan, four (57.1%) developed progressive peritoneal carcinomatosis, and one (14.3%) showed new retroperitoneal nodal metastases. Each of the four patients with clinical response had received a different trientine dosage level (300, 900, 1,500, and 1,800 mg). All four patients with clinical response had serous histology and were allocated to partially platinum-sensitive group. Three other patients (18.8%) achieved SD for ≥4 months. Subgroup analysis showed the clinical benefit rate was 33.3 and 50.0% in the platinum-resistant group and the partially platinum-sensitive group, respectively (Table 6).

**Table 6:** Treatment response based on RECIST 1.1 criteria and GCIC CA125 criteria.

**Response**	**All patients *N* (%)**	**Partially platinum-sensitive*N* (%)**	**Platinum-resistant/platinum-refactory*N* (%)**
Complete response	2 (12.5)	2 (20.0)	0 (0)
Partial response	2 (12.5)	2 (20.0)	0 (0)
Stable disease	3 (18.8)	1 (10.0)	2 (33.3)
Progressive disease	9 (56.3)	5 (50.0)	4 (66.7)
Not evaluable	2	–	–

*RECIST 1.1, the Response Evaluation Criteria in Solid Tumors version 1.1; GCIC CA125, the Gynecologic Cancer Intergroup definition for carbohydrate antigen 125 progression*.

The overall response rate was 40.0% in the latter group. The survival curves stratified by progression-free interval of 6 months were illustrated in Supplementary Figure 2. For all 16 patients, the median PFS duration was 4.6 months [95% confidence interval (CI), 0–11.3 months], and the median OS was 14.4 months (95% CI, 0–34.2 months). Compared with the platinum-resistant group, the median PFS and OS duration was greater in the partially platinum-sensitive group (7.9 vs. 2.3 months, *P* = 0.518; 14.4 vs. 5.4 months, *P* = 0.326, respectively). However, there was no statistical significance in survival differences between these groups.

## Discussion

The present study provides clinical evidence supporting the safety of the combination regimen of PLD plus platinum plus oral trientine, administered 7 days prior to the other chemotherapy agents, in patients with EOC/TC/PPSC. Additionally, this is the first study to describe the PK parameters of TETA and its two metabolites in EOC patients. According to a previous report, the neurologic AEs caused by D-penicillamine may be irreversible (24). Hence, we selected the less neurotoxic trientine for our trial. This agent is most commonly associated with headache at daily doses ≥600 mg (24), although other AEs such as iron deficiency (26), thrombocytopenia (27), anemia (28, 29), liver toxicity (30), or dizziness (13) have been reported. In this study, however, we rarely observed significant headaches, but did observe grade 2 dizziness at a trientine dose level of 1,200 mg; this was reversible when the daily dose was reduced. No treatment-related deaths or DLT were observed. Thus, this copper-lowering combination approach was safe and well-tolerated even up to daily trientine doses of 1,800 mg.

Previous reports have described elevated serum concentrations of copper and ceruloplasmin in patients with gynecological cancers (31, 32). Notably, Fu et al. reported that the combination of trientine and carboplatin exhibited antitumor activity, especially in patients who experienced reductions in copper and/or ceruloplasmin levels (15). However, in our analysis of serum and urine biomarkers associated with a copper-lowering approach, we observed little correlation between the baseline or nadir levels of these biomarkers and clinical responses or patient outcomes. Instead, only a high baseline serum iron level and low serum copper level following three cycles of copper-lowering therapy might differentiate subgroups of patients with different clinical responses. Moreover, as all of our patients had nadir ceruloplasmin levels >15 mg/dL, we did not identify any survival advantage among patients with low ceruloplasmin levels. Reasons for this discrepancy are unknown, but it may be due to differences in ethnicities or in proportions of histology between these studies. A large-scale study may be needed to examine the applicability of these biomarkers.

For TETA at a low dose of trientine (600 mg/day), WD patients and healthy volunteers exhibited T_max_ in the range of 1.6–3.5 and 1.1–2.0 h and C_max_ in the range of 0.5–14.0 and 0.7–1.0 mg/L, respectively (13, 24, 25). Healthy volunteers showed AUC_0−24_ values of TETA in the range of 2.9–4.2 mg × h/L. For TETA at a high dose of trientine (≥900 mg/day), WD patients and healthy volunteers showed T_max_ in the range of 1.6–3.5 and 0.8–2.0 h and C_max_ in the range of 0.8–14.0 and 1.6–5.6 mg/L, respectively. Healthy volunteers showed AUC_0−24_ values of TETA in the range of 10.0–20.0 mg × h/L. Our results indicate fast absorption and rapid elimination of the parent compound after oral trientine administration in EOC patients. Similar to polyamines, TETA may be taken up by the intestines or transported across the biological membrane into cells via the transporter glypican-1 (25). EOC patients may express high levels of this transporter. Furthermore, different levels of the major acetylation enzymes (N-acetyltransferase or spermidine/spermine acetyltransferase) may be responsible for the variations in acetylation of TETA in different populations (13, 25).

Patients with platinum-resistant/refractory EOC generally exhibit poor tumor responses to rechallenge with platinum-containing chemotherapy. Therefore, efforts have been made to identify drugs that overcome platinum resistance (4, 5, 10, 12, 15, 16), alternative chemotherapies (17–19, 33), dose-dense schedules (34), the joint use of anti-angiogenic agents (35), and synthetic lethality (36, 37). Fu et al. reported combination treatment with trientine and carboplatin in recurrent EOC patients who had previously received a median of four lines of chemotherapy (15). The overall response and clinical benefit rates were 12.5 and 37.5%, respectively (15). In our study, we adopted the two aforementioned approaches with chemotherapy and studied its feasibility and safety profiles prior to a further study, which aims at studying the related treatment outcomes.

Although there were discrepancies in the inhibitory effects of our combination therapy between the xenograft animal study and the platinum-resistance/-refractory group, we demonstrated modest antitumor activity in the partially platinum-sensitive subgroup. The CALYPSO phase III trial focused on the treatment effects of carboplatin plus PLD in partially platinum-sensitive EOC, reporting an overall response rate of 39% (38). Benefits in treatment response or prognosis in our study did not appear to be caused by the addition of trientine to carboplatin and PLD. This could be due to only 10 such patients enrolled within the limited study period; alternatively, it could be influenced by the distribution in ethnicity or CCC-dominant histology in our study. Therefore, efficacy could not be determined.

In conclusion, this study clearly demonstrated the safety and PK profiles of trientine in a combination therapy of trientine plus carboplatin and PLD. While the sample size of our current study was relatively small and only Asian patients were recruited, this is the largest study to date to investigate trientine in combination with platinum-based chemotherapy for patients with platinum-resistant or early relapsed EOC/TC/PPSC. Serum levels of iron and copper were identified as the biomarkers to reflect clinical benefits in this study. A future phase II trial should assess treatment advantages of this combination in a larger sample of patients with early-relapsed EOC and latent platinum-resistance.

## Data Availability

The raw data supporting the conclusions of this manuscript will be made available by the authors, without undue reservation, to any qualified researcher.

## Ethics Statement

The clinical research protocol was approved by the National Cheng Kung University Hospital (NCKUH) Institutional Review Board. Each patient provided written consent in accordance with the Declaration of Helsinki.

Our study complied with the National Centre for the Replacement, Refinement and Reduction of Animals in Research, and were approved by the Institutional Animal Care and Use Committee of National Cheng Kung University.

This study followed the Animal Research: Reporting of *in vivo* Experiments guidelines and the National Institutes of Health guide for the care and use of Laboratory animals (NIH Publications No. 8023, revised 1978).

## Contribution to the Field Statement

Epithelial ovarian cancer (EOC) is the most lethal gynecological cancer worldwide. Tubal cancer and primary peritoneal serous carcinoma are thought to form in the same tissue and are treated the same way. Most of those patients with advanced stage will eventually progress after treatment, complicated by platinum resistance. The copper-lowering agent trientine has been shown to re-sensitize platinum-resistant cancer cells by enhancing the hCtr1-mediated uptake of platinum. Moreover, no investigation has reported the pharmacokinetics of trientine in cancer patients. Drawing on discoveries in preclinical studies, animal models and the clinical study in individuals with EOC, we propose to provide *in vivo* evidence that a combination of pegylated liposomal doxorubicin (PLD) plus platinum plus the oral trientine, administered 7 days prior to the other chemotherapy agents, is a safe and tolerable treatment in those patients. Our cancer patients exhibit greater absorption and faster elimination after oral trientine when compared with healthy volunteers or Wilson's disease patients in the literature. Biomarkers may be used to reflect responses to combined PLD-trientine-carboplatin chemotherapy. This combination therapy is well-tolerated as no treatment-related deaths or dose-limiting toxicities were observed. The efficacy of this approach should be further verified by conducting a large-scale clinical trial.

## Author Contributions

Y-FH, Y-MC, Y-SL, and C-YC conducted research, acquired data, and interpreted the results. Y-FH and P-YW performed the statistical analysis. MK and C-YC provided administrative support. All authors participated in the conception or design of the present study, drafted and revised the article for important intellectual content, and approved the final submitted version of the manuscript.

### Conflict of Interest Statement

Y-FH, MK, and C-YC obtained two patents (US PATENT no. US9770462B2 and TAIWAN PATENT no. I604847). The remaining authors declare that the research was conducted in the absence of any commercial or financial relationships that could be construed as a potential conflict of interest.

## Supplementary Material

Supplementary Figure 1 Xenograft animal model. Four mouse groups were treated with different combinations of chemotherapeutic agents (anthracycline/carboplatin) plus trientine, which was administered concomitantly with or 7 days prior to chemotherapy. **(A)** Tumor formation (red circle). **(B)** Tumor size. **(C)** The change of mean body weight among each group.

Supplementary Figure 2 Overall survival (OS) and progression-free survival (PFS). Kaplan-Meier curves stratified by progression-free interval of 6 months, tested by log-rank test. The median PFS duration was 7.9 and 2.3 months, and the median OS was 14.4 and 5.4 months for partially platinum-sensitive group and platinum-resistant group, respectively.

Supplementary Table 1 Animal study treated with different chemotherapeutic combinations (anthracycline/ carboplatin) plus trientine given concomitantly with chemotherapy or 7 days prior to chemotherapy.
