# Supplementary figures and images for: A Dose Escalation Study of Trientine Plus Carboplatin and Pegylated Liposomal Doxorubicin in Women With a First Relapse of Epithelial Ovarian, Tubal, and Peritoneal Cancer Within 12 Months After Platinum-Based Chemotherapy
Source: Front Oncol. 2019 May 24;9:437. doi: 10.3389/fonc.2019.00437 (PMC6544081; doi:10.3389/fonc.2019.00437)

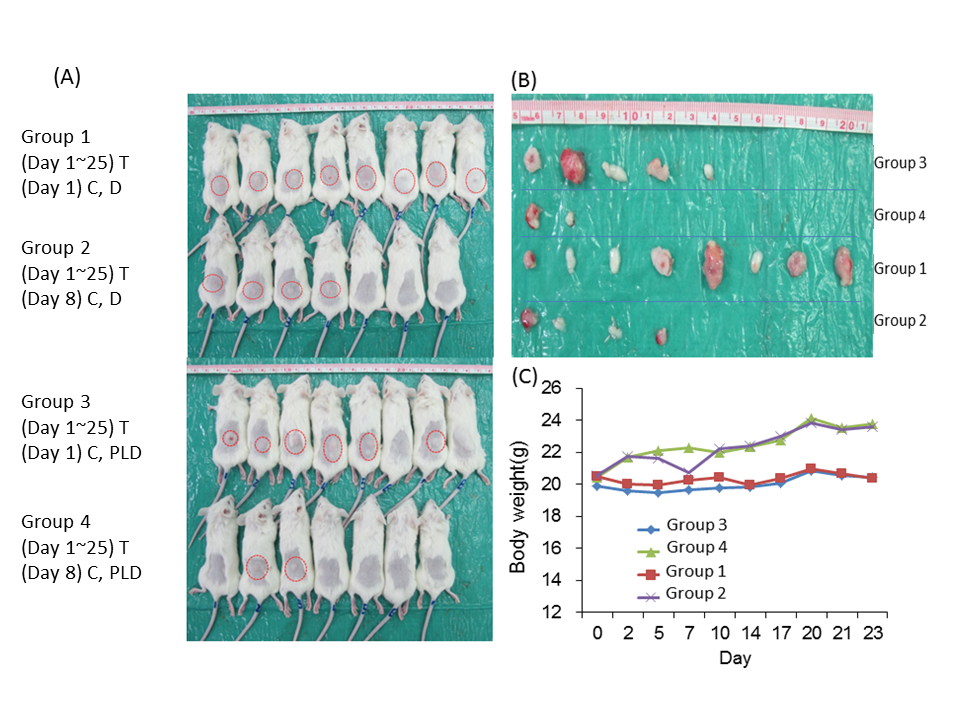

Supplement: Supplementary Figure 1 — Xenograft animal model. Four mouse groups were treated with different combinations of chemotherapeutic agents (anthracycline/carboplatin) plus trientine, which was administered concomitantly with or 7 days prior to chemotherapy. (A) Tumor formation (red circle). (B) Tumor size. (C) The change of mean body weight among each group. [file Image_1.TIF]

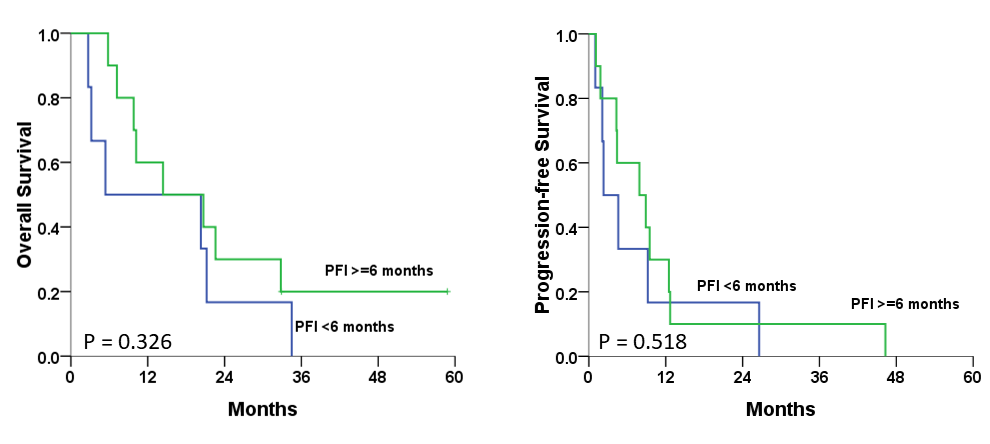

Supplement: Supplementary Figure 2 — Overall survival (OS) and progression-free survival (PFS). Kaplan-Meier curves stratified by progression-free interval of 6 months, tested by log-rank test. The median PFS duration was 7.9 and 2.3 months, and the median OS was 14.4 and 5.4 months for partially platinum-sensitive group and platinum-resistant group, respectively. [file Image_2.TIF]
