# Supplementary material for: A Dose Escalation Study of Trientine Plus Carboplatin and Pegylated Liposomal Doxorubicin in Women With a First Relapse of Epithelial Ovarian, Tubal, and Peritoneal Cancer Within 12 Months After Platinum-Based Chemotherapy
Source: Front Oncol. 2019 May 24;9:437. doi: 10.3389/fonc.2019.00437 (PMC6544081; doi:10.3389/fonc.2019.00437)
Supplement: Supplementary Table 1 — Animal study treated with different chemotherapeutic combinations (anthracycline/ carboplatin) plus trientine given concomitantly with chemotherapy or 7 days prior to chemotherapy. [file Table_1.DOCX]

**Supplementary Table 1. Animal study treated with different chemotherapeutic combinations (anthracycline/ carboplatin) plus trientine given concomitantly with chemotherapy or 7 days prior to chemotherapy.**

| **Group** | **Drugs** | **N** | **Tumor volume (mm^3^)** | **P** | **Tumor formation rate** | **P** |
| --- | --- | --- | --- | --- | --- | --- |
| **1** | T (day 1~25)  C+D (day 1) | 8 | 522.9 ± 72.4 | ̶ | 8/8  (100%) | ̶ |
| **2** | T (day 1~25),  C+D (day 8) | 7 | 210.5 ± 65.4 | 0.027 | 4/7  (57.2%) | 0.077 |
| **3** | T (day 1~25)  C+PLD (day 1) | 8 | 366.7 ± 104.9 | 0.245 | 6/8  (75.0%) | 0.467 |
| **4** | T (day 1~25)  C+PLD (day 8) | 7 | 149.7 ± 71.5 | 0.068 | 2/7  (28.6%) | 0.007 |

Data is presented as mean ± standard deviation.

T, Trientine; D, doxorubicin; C, carboplatin; PLD, pegylated liposomal doxorubicin.
